# Comparison of the characteristics, morbidity and mortality of COVID-19 between first and second/third wave in a hospital setting in Lombardy: a retrospective cohort study

**DOI:** 10.1007/s11739-022-03034-5

**Published:** 2022-07-09

**Authors:** Francesca Leidi, Gianluca Edoardo Mario Boari, Ottavio Scarano, Benedetta Mangili, Giulia Gorla, Andrea Corbani, Beatrice Accordini, Federico Napoli, Chiara Ghidelli, Giulia Archenti, Daniele Turini, Michele Saottini, Vittoria Guarinoni, Giulia Ferrari-Toninelli, Francesca Manzoni, Silvia Bonetti, Giulia Chiarini, Paolo Malerba, Federico Braglia-Orlandini, Gianluca Bianco, Cristina Faustini, Claudia Agabiti-Rosei, Carolina De Ciuceis, Damiano Rizzoni

**Affiliations:** 1 Division of Medicine, Spedali Civili di Brescia, Montichiari, Brescia Italy; 2 Department of Clinical and Experimental Sciences, University of Brescia, c/o 2a Medicina Spedali Civili Di Brescia, Piazza Spedali Civili 1, 25100 Brescia, Italy

**Keywords:** COVID-19, SARS-CoV2, Prognostic factors, Waves, Mortality

## Abstract

Coronavirus disease 2019 (COVID-19) represents a major health problem in terms of deaths and long-term sequelae. We conducted a retrospective cohort study at Montichiari Hospital (Brescia, Italy) to better understand the determinants of outcome in two different COVID-19 outbreaks. A total of 634 unvaccinated patients admitted from local emergency room to the Internal Medicine ward with a confirmed diagnosis of SARS-CoV-2 infection and a moderate-to-severe COVID-19 were included in the study. A group of 260 consecutive patients during SARS-CoV-2 first wave (from February to May 2020) and 374 consecutive patients during SARS-CoV-2 2nd/3rd wave (from October 2020 to May 2021) were considered. Demographic data were not significantly different between waves, except a lower prevalence of female sex during first wave. Mortality was significantly higher during the 1^st^ wave than in the following periods (24.2% vs. 11%; *p* < 0.001). Time from symptoms onset to hospital admission was longer during first wave (8 ± 6 vs. 6 ± 4 days; *p* < 0.001), while in-hospital staying was significantly shorter (10 ± 14 vs. 15 ± 11 days; *p* < 0.001). Other significant differences were a larger use of corticosteroids and low-molecular weight heparin as well less antibiotic prescription during the second wave. Respiratory, bio-humoral and X-ray scores were significantly poorer at the time of admission in first-wave patients. After a multivariate regression analysis, C-reactive protein and procalcitonin values, % fraction of inspired oxygen on admission to the Internal Medicine ward and length of hospital stay and duration of symptoms were the strongest predictors of outcome. Concomitant anti-hypertensive treatment (including ACE-inhibitors and angiotensin-receptor blockers) did not affect the outcome. In conclusion, our data suggest that earlier diagnosis, timely hospital admission and rational use of the therapeutic options reduced the systemic inflammatory response and were associated to a better outcome during the 2nd/3rd wave.

## Introduction

Coronavirus disease 2019 (COVID-19) is a respiratory tract infection caused by SARS-CoV-2, a novel coronavirus that was first recognised in Wuhan, China, in December 2019. Since then, cases have been spreading worldwide in an impressive high rate, so that a pandemic and Public Health Emergency of International Concern has been declared by WHO on 30 January 2020 [1].

Besides the compelling need to understand the biological pathways underlying the virulence and pathogenicity of SARS-CoV-2 to develop new treatments, one of the most significant and challenging issues emerging from 2 years of pandemic is the dramatic difference in mortality rates between the first and the subsequent COVID-19 waves. Italy indeed had the highest number of recorded COVID-19 deaths in Europe during the first wave, from February 2020 to May 2020. In particular, Lombardy (Italy) was the first European region hit by the SARS-CoV-2 pandemics [2] and one of the most affected regions worldwide during the first wave in term of death toll and long-term sequelae [3].

In Italy after a summer defervescence with relatively low infection incidence [4], a second wave began in late August and peaked in late October 2020, then continued with the third wave till May 2021, without a clear separation between them, such that they are considered as a single identity (2nd/3rd wave) in the present study. The vaccination campaign started in Italy on 27th December 2020.

Therefore, a single-centered retrospective cohort study was conducted at the Montichiari Hospital, which is a tertiary health-care center in Brescia (Lombardy) designated as a COVID-19 hub by the Italian health authorities, to better define the characteristics of the unvaccinated populations affected by COVID-19 in the different waves and to identify the predictors of mortality.

### Patients and methods

A total of 634 unvaccinated patients admitted from the emergency room of the Montichiari Hospital (Brescia, Italy), to the Department of Internal Medicine with a confirmed diagnosis of SARS-CoV-2 infection by a positive real-time reverse-transcription polymerase chain reaction on a nasopharyngeal swab [5] were included in the present study. A group of 260 consecutive patients during SARS-CoV-2 first wave (from February 2020 to May 2020) and 374 consecutive patients during SARS-CoV-2s/third wave (from October 2020 to May 2021) were considered. Patients transferred from other health facilities were excluded from the present analysis.

All patients included in the present study were not vaccinated for COVID-19.

Demographic data, comorbidities, ongoing treatment and bio-humoral, respiratory and haemodynamic data were recorded and compared between the two main outbreaks to better understand the COVID-19 characteristics and mortality rate.

In particular, for every patient, an arterial blood gas analysis and a chest X-ray were routinely performed at ER admission. After the first assessment in ER, patients according to their clinical conditions were treated with appropriate oxygen supplementation, delivered with nasal prongs (high flow nasal-cannula were not available yet at Montichiari Hospital), Venturi mask or non-invasive mechanical ventilation, and then transferred to the Internal Medicine ward. At admission in the Internal Medicine ward, vital parameters (blood pressure, heart rate, O_2_ saturation measured by a finger pulse oximeter) and venous blood test, including complete blood count, CRP, procalcitonin, ferritin, D-dimer, INR, aPTT, were collected.

### Statistical analysis

Data were analyzed with SPSS version 25.0 (Chicago, IL, USA). We report categorical variables as percentages (%) and continuous variables as means ± standard deviation when data were normally distributed, and as medians and interquartile range when data were not normally distributed (i.e. lymphocytes, procalcitonin, ferritin, D-dimer values). Statistical significance between groups was assessed by means of Student’s *t* test for quantitative variables and *χ*^2^ test for qualitative ones, by means of one-way analysis of variance (ANOVA) or by Mann–Whitney *U* test when appropriate. A multivariate logistic regression analysis was also performed to identify predictors of mortality (stepwise forward); then receiver operating characteristic (ROC) curves were calculated to assess the sensibility and specificity of the possible mortality determinants. The statistical significance threshold was set at *p* < 0.05.

### Results

Main demographic data (age, gender, comorbidities, duration of symptoms before hospitalization, length of hospital stay and death rate) and previous drug treatment at admission are reported, respectively, in Tables 1 and 2. There was no significant difference between the populations of the 1st and the 2nd/3rd wave, except a lower prevalence of female sex during first one, as shown in Tables 1 and 2. Mortality rate was significantly lower during the latter period (24.2% vs. 11.0%; *p* < 0.001). Time from symptoms onset to hospital admission was longer during the first wave (8 ± 6 vs. 6 ± 4 days; *p* < 0.001), while hospital stay was significantly lower (10 ± 14 vs. 15 ± 11 days; *p* < 0.001). No significant differences between COVID-19 and hospital-staying-related complications were detected between the two populations studied, except a higher incidence of concomitant atypical bacteria pneumonia during the 1st wave (diagnosis confirmed by serological antibody test). There was a trend to significance in sepsis higher incidence in the first wave (*p* = 0.73), possibly due to both worse overall conditions of patients at admission and administration of higher doses of dexamethasone (as suggested by expert consensus at the beginning of pandemic).

**Table 1:** Demographic data

	First wave	Second/third wave	Significance
Total patients (#)	260	374	
Dead (#, %)	63 (24.2%)	41 (11.0%)	***
Age (years)	71 ± 13	69 ± 15	NS
Gender (males/females, % males)	175/85 (67.3%)	207/167 (55.3%)	**
Diabetes (#, %)	64 (24.6%)	103 (27.5%)	NS
Hypertension (#, %)	145 (55.8%)	239 (63.9%)	NS
Heart disease (#, %)	84 (42.3%)	146 (39.0%)	NS
COPD (#, %)	32 (12.3%)	33 (8.82%)	NS
Smoke (actual or previous) (#, %)	37 (14.2%)	51 (13.6%)	NS
Weight (kg)	77.3 ± 14.5	79.1 ± 17.6	NS
Cancer (#, %)	10 (3.85%)	20 (5.35%)	NS
Duration of symptoms before hospitalization (days)	8 ± 6	6 ± 4	***
Duration of hospitalization (days)	10 ± 14	15 ± 11	***

*COPD* chronic obstructive pulmonary disease, # total number

**p < 0.01, ***p < 0.001

**Table 2:** Previous therapy and respiratory and radiologic parameters at hospital admission

	First wave (*n* = 260)	Second/third wave (*n* = 374)	Significance
**Previous therapy**			
ACE-inhibitors (#,%)	48 (18.5%)	90 (24.1%)	NS
Angiotensin-receptor blockers (#,%)	49 (18.8%)	57 (15.2%)	NS
Statins (#,%)	71 (27.3%)	105 (28.1%)	NS
Anti-platelet agents (#,%)	71 (27.3%)	94 (25.1%)	NS
Steroids (#,%)	10 (3.84%)	26 (6.95%)	NS
Anticoagulants (#,%)	26 (10%)	98 (26.2%)	NS
**Hemogasanalysis at the arrival in ER**			
apH	7.48 ± 0.05	7.46 ± 0.04	***
apO_2_ (mm Hg)	60.3 ± 18.4	65.3 ± 12.3	***
apCO_2_ (mm Hg)	34.5 ± 6.6	34.6 ± 5.4	NS
aSpO_2_%	89 ± 10.8	92.1 ± 7.3	***
FiO_2_%	25.7 ± 14.4	23 ± 7	**
pO_2_/FiO_2_	262 ± 90	297 ± 65	***
**Chest X-ray at ER**			
COVID Brixia score	7.9 ± 4	6 ± 4	***

*ACE* angiotensin converting enzyme, *#* total number, *ER* emergency room, *apH* arterial pH, *apO*_*2*_ arterial partial pressure of oxygen, *apCO*_*2*_ arterial partial pressure of carbon dioxide, *aSpO*_*2*_ arterial oxygen saturation, *FiO*_*2*_ fraction of inspired oxygen in the arterial blood sample, *NS* not statistically significant

*p < 0.05, **p < 0.01, ***p < 0.001

Respiratory, bio-humoral and radiologic data were significantly worse at the time of admission in first-wave patients and major significant differences between the 1st and 2nd/3rd waves are reported in Tables 2 and 3.

**Table 3:** Hemodynamic, respiratory and bio-humoral parameters at the arrival in the Internal Medicine ward, in-hospital treatment and main complications

	First wave (*n* = 260)	Second/third wave (*n* = 374)	Significance
**Vital parameters at the arrival in the Internal Medicine ward**			
Systolic blood pressure (mmHg)	129 ± 21	133 ± 21	*
Diastolic blood pressure (mmHg)	75 ± 11	77 ± 13	NS
Mean blood pressure (mmHg)	93 ± 15	95 ± 14	**
Heart rate (beats/min)	86 ± 16	84 ± 15	NS
SpO_2_%	94 ± 4	95 ± 3	NS
FiO_2_%	40 ± 25	26 ± 10	***
Nasal prongs	15 ± 0.48	25 ± 0.50	NS
Venturi mask	124 ± 0.50	112 ± 0.49	***
NIV	8 ± 0.24	6 ± 0.22	NS
Temperature (°C)	37.7 ± 1	37 ± 0.9	***
**Blood tests at arrival in the Internal Medicine ward**			
Hemoglobin (g/dl)	12.8 ± 1.8	13 ± 1.9	NS
White blood cells (#/mm^3^)	4.5 ± 3.5	6.8 ± 8.6	***
Granulocytes (#/mm^3^)	3.2 ± 2.3	4.6 ± 2.8	***
Monocytes (#/mm^3^)	0.5 ± 0.6	0.6 ± 1.5	NS
Lymphocytes (#/mm^3^)	1.3 ± 2	1.7 ± 6.8	NS
Platelets (#/mm^3^)	211 ± 84	202 ± 79	NS
CRP (mg/l)	99.5 ± 76.7	55.9 ± 56.4	***
Procalcitonin (ng/ml)	1.1 ± 3.6	0.4 ± 1.5	*
Ferritin (µg/l)	983 ± 1224	760 ± 742	NS
D-dimer (ng/ml)	1629 ± 6664	1028 ± 1366	NS
INR	1.3 ± 0.3	1.1 ± 0.6	***
aPTT (s)	32.7 ± 4	34.1 ± 7.6	*
**Main treatments**			
Steroids (#, %)	81 (31.1%)	344 (92.0%)	***
Oxygen (any device. any flow) (#)	207	302	NS
LMWH prophylactic standard dose	94	119	*
LMWH prophylactic high dose (#)	31	164	***
LMWH anticoagulant high dose (#)	25	38	NS
Antibiotics (#, %)	222 (85.4%)	283 (75.7%)	***
Main complications			
Pulmonary thromboembolism/CT (#, %)	16/41 (39.0%)	25/101 (24.8%)	NS
Sepsis (#, %)	18 (6.92%)	12 (3.21%)	NS
Atypical bacterial infection (#, %)	31 (11.9%)	22 (5.88%)	**
Delirium (#, %)	19 (7.31%)	30 (8.02%)	NS

*SpO*_*2*_ peripheral oxygen saturation, *FiO*_*2*_ fraction of inspired oxygen, *NIV* non-invasive mechanic ventilation, *CRP* C-reactive protein, *INR* international normalized ratio, *aPTT* activated partial thromboplastin time, # total number, *LMWH* low molecular weight heparin, *CT* computerized tomography, *NS* not statistically significant

*p < 0.05, **p < 0.01, ***p < 0.001

Moreover, concerning the respiratory parameters of the arterial blood test collected at the entrance in ER, in the 2nd/3rd wave, a lower fraction of inspired oxygen (FiO_2_) was needed, so that a higher P/F (pO_2_/FiO_2_**)** ratio, was observed. The Brixia score, a radiologic score proposed by Borghesi and Maroldi [6], whose prognostic value was clearly demonstrated [7–10], was used to evaluate the severity index of the lung involvement in SARS-CoV-2 pneumonia. A more severe lung involvement was observed in the first wave patients (Table 2), as higher values of Brixia radiologic score were recorded. Similarly in the first wave a larger use of oxygen supplementation with Venturi mask in the ER as well as a higher fraction of inspired oxygen (FiO_2_) recorded at the entrance of the Internal Medicine ward were observed. In particular, in the first wave cohort (*n* = 260) 8 patients were treated with non-invasive mechanical ventilation in the ER, 124 with Venturi mask and 15 with nasal prongs. In the subsequent outbreaks (*n* = 374), six patient underwent non-invasive mechanical ventilation, 112 needed a Venturi mask and 25 nasal prongs (Table 3).

Regarding bio-humoral data, C-reactive protein (CRP), and procalcitonin serum levels were significantly higher during first wave on hospital admission.

Other significant differences concern drug treatment during hospital stay: a larger and more standardized use of corticosteroids and low-molecular weight heparin (LMWH) prophylactic dose, together with less antibiotic prescription were observed during the second wave (Table 3), in agreement with the International Guidelines published at that time [11]. Previous and concomitant anti-hypertensive treatment, including angiotensin converting enzyme (ACE) inhibitors and angiotensin-receptor blockers did not affect the outcome (Table 2).

A multivariate regression analysis was performed in the two cohorts separately (Tables 4, 5), considering survival as independent variable; dependent variables were duration of symptoms before hospitalization, duration of hospitalization, age, gender, respiratory parameters (mainly FiO_2_ at admission in the Internal Medicine ward), bio-humoral parameters (haemoglobin, lymphocytes, creatinine, CRP), comorbidities (heart disease, hypertension, diabetes, chronic obstructive pulmonary disease—COPD, active or previous smoke). Comorbidities were included as potential confounders, as they were clearly demonstrated to influence the risk of mortality [12, 13].

**Table 4:** Multivariate regression with survival as independent variable in the first wave

Alive	Coef	Std. err	*z*	*p* >| *z* |	95% Conf.	Interval
Duration of symptoms before hospitalization (days)	0.2197149	0.1037386	2.12	0.034	0.0163909	0.4230388
Duration of hospitalization (days)	0.2083777	0.0824275	2.53	0.011	0.0468227	0.3699327
Age	− 0.1073714	0.0498325	− 2.15	0.031	− 0.2050413	− 0.0097015
Gender	0.1009582	0.9382182	0.11	0.914	− 1.737916	1.939832
Heart disease	− .377227	0.9382182	− 0.41	0.684	− 2.196796	1.442342
Hypertension	− 3.263427	1.744362	− 1.87	0.061	− 6.682314	0.1554605
Diabetes	0.8186146	1.051425	0.78	0.436	− 1.232141	2.87937
COPD–asthma	− 2.050069	1.203139	− 1.70	0.088	− 4.408178	0.3080391
FiO_2_ (%)	− 1.857554	1.908401	− 0.97	0.330	− 5.597952	1.882844
Haemoglobin at admission (g/dl)	0.1092671	.2486386	0.660	0.660	− 0.3780556	0.5965898
Lymphocytes at admission (#/mm^3^)	− 0.5455914	.603866	0.366	0.366	− 1.729147	0.6379642
Creatinine at admission (mg/dl)	− 0.7286528	.7462173	0.329	0.329	− 2.191212	0.7339061
CRP at admission (mg/l)	− 0.0039497	.0051104	0.440	0.440	− 0.0139659	0.0060664
Smoke	− 0.4167725	.554533	0.4522	0.452	− 1.503637	0.6700923
Logistic regression				Number of obs	= 129	
Log likelihood = − 25.169263				LR chi2	= 60.95	
				Prob > chi2	= 0.0000	
				Pseudo *R*^2^	= 0.5477	

*COPD* chronic obstructive pulmonary disease, *FiO*_*2*_ inspired fraction of oxygen, *CRP* protein C reactive

**Table 5:** Multivariate regression with survival as independent variable in the second/third wave

Alive	Coef	Std. err	*z*	*p* >| *z* |	95% Conf.	Interval
Duration of symptoms before hospitalization (days)	0.2440133	0.0885828	2.75	0.006	0.0703942	0.4176324
Duration of hospitalization (days)	− 0.035455	0.0173108	− 2.05	0.041	− 0.0694031	− 0.0015069
Age	− 0.0241358	0.025261	− 0.96	0.339	− 0.0736464	0.0253749
Gender	− 0.1828182	0.5953193	− 031	0.759	− 1.349623	0.9839862
Heart disease	− 0.8266688	0.7164333	− 1.15	0.249	− 2.230852	0.5775147
Hypertension	0.8525657	0.6799574	1–25	0.210	− 0.4801264	2.185258
Diabetes	− 0.2872164	0.6758516	− 0.42	0.671	− 1.611861	1.037428
COPD–asthma	0.1828464	0.8412785	0.22	0.828	− 1.466029	1.831722
FiO_2_ (%)	− 0.7682457	3.205417	− 0.24	0.811	− 7.050749	5.514257
Haemoglobin at admission (g/dl)	0.3260838	0.1424997	2.29	0.022	0.0467895	0.605378
Lymphocytes at admission (#/mm^3^)	− 0.037123	0.0428692	− 0.87	0.387	− 0.121145	0.046899
Creatinine at admission (mg/dl)	− 0.2216111	0.247152	− 0.90	0.370	− 0.7060201	0.262798
CRP at admission (mg/l)	0.0691777	0.0046722	− 2.50	0.012	− 0.0208559	− 0.0025414
Smoke	0.7710217	0.4273573	0.16	0.871	− 0.7684272	0.9067826
Logistic regression				Number of obs	= 261	
Log likelihood = − 25.169263				LR chi2	= 59.56	
				Prob > chi2	= 0.0000	
				Pseudo *R*^2^	= 0.3614	

*COPD* chronic obstructive pulmonary disease, *FiO*_*2*_ inspired fraction of oxygen, *CRP* protein C reactive

The duration of hospitalization strongly influenced the risk of dying from COVID-19 in both periods considered. However, interestingly enough, the duration of the hospital admission showed a direct, positive correlation with the chance of survival during the first wave; while an inverse, negative correlation was observed in the second/third wave.

The multivariate regression analysis was repeated excluding comorbidities as dependent variables, and including the followings variables that differ significantly between the two periods: duration of symptoms before hospitalization, duration of hospitalization, body temperature, systolic blood pressure at admission, respiratory parameter at admission: FiO_2_ delivered in ER, arterial pH (apH), arterial partial pressure of oxygen (apO_2_), arterial oxygen saturation (aSpO_2_), P/F measured subsequently, bio-humoral parameters at admission (ferritin, CRP, procalcitonin, partial thromboplastin time) and Brixia radiologic score at admission.

When only the first wave was considered, the following variables were significant predictors of outcome: duration of hospitalization (*p* = 0.019), procalcitonin levels (*p* = 0.002), apH (*p* = 0.010) and body temperature (*p* = 0.042); when only the second/third wave were considered, the following variables entered the model: FiO_2_ (*p* < 0.001), procalcitonin (*p* < 0.001) and CRP (*p* = 0.008) levels, Finally, when both first and second/third waves were considered, the following variables were included in the model: procalcitonin (*p* = 0.002), CRP (*p* = 0.012), FiO_2_ (*p* < 0.001), duration of hospitalization (*p* = 0.013) and duration of symptoms (*p* = 0.033).

CRP, a biomarker of inflammation, was, therefore, related to the mortality when both cohorts were studied together. Similarly, the fraction of inspired oxygen (FiO_2_) needed at the hospital admission was the only respiratory parameter strongly associated to the mortality when both periods were considered together. Also, procalcitonin circulating levels and duration of symptoms and of hospitalization were significant predictors of mortality in this setting. These analyses were carried out excluding patients with established diagnosis of concomitant bacterial infection (e.g. microbial isolation in the blood or urine samples or in the respiratory materials [sputum culture, tracheal aspirates or bronchoalveolar lavage], serum antibodies for atypical bacteria pneumonia, pneumococcal and legionella urinary antigen tests, etc.).

**Fig. 1:**
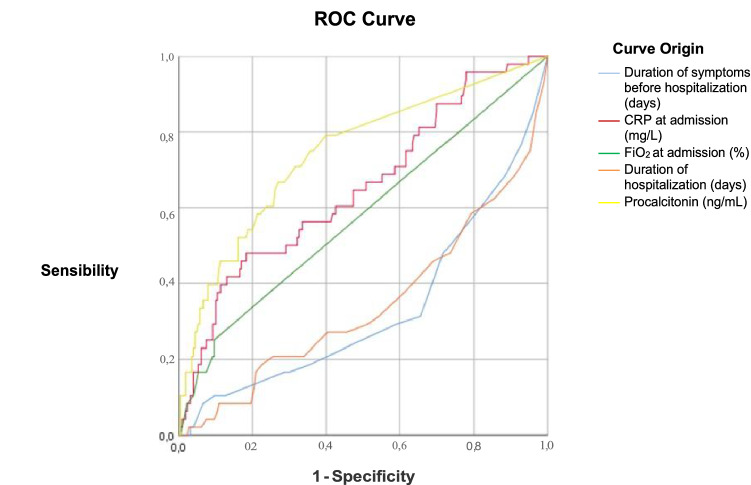
Receiver operating characteristic (ROC) curves. Area under the curve: duration of symptoms: 0.333, duration of hospital admission: 0.346, FiO_2_ at entry: 0.577, CRP serum levels: 0.652, procalcitonin plasma levels: 0.745

Therefore, to better assess the sensibility and specificity of CRP dosage and FiO_2_ at the admission as possible mortality predictors in COVID-19 in an unvaccinated population, a ROC curve analysis was conducted, including all patients (Fig. 1). By measuring the area under the ROC curve, it resulted that the most specific and sensible predictor of mortality is represented by procalcitonin circulating levels, followed by the duration of hospital admission and FiO_2_ (Fig. 1).

## Discussion

This study evaluated the characteristics, morbidity and short-term outcome of a population of 634 COVID-19 patients hospitalised in Montichiari Hospital during the first wave and then during the second/third wave. In the present study, the mortality rate observed during the first wave (24%) is in agreement with what observed by Giacomelli et al. [14] (about 20%) in Italy, by Docherty et al. [15] (26%) in the United Kingdom, by Richardson et al. [16] in New York City area (21%). This mortality rate is higher if compared to the 14% estimated by Wu et al. [17] in the early pandemic in China. The death toll observed in second/third wave is significantly lower (11%) than that of the early stage of the pandemic; these data are confirmed by similar results in USA and in main European countries (except Germany and Sweden), as demonstrated by James et al. [18], Vinceti et al. [4] and Borghesi et al. [19] in Italy.

Among the factors that might explain such a difference in mortality, a shorter duration of symptoms before hospital admission and better respiratory parameters might have played a role, suggesting a timelier admission, which occurred on average before the progression of the lung involvement to a more severe extent.

Moreover, it should be noted that the impact of COVID-19 decreased progressively from 1st wave to 2nd–3rd wave, although none of these patients was vaccinated.

Preliminary data from our ward suggest that during the 4th wave (January–February 2022), the impact of the disease was milder, both in the vaccinated population (around 80%, *n* = 55) and also in the “no vax” population (around 20% *n* = 15), with only a couple of deaths safely ascribable to COVID-19 observed.

Therefore, such an impressive decrease in mortality in a non-vaccinated population may be fully explained not only by the factors reported above (i.e. early diagnosis and hospitalization and improvement of the hospital treatment and care) but maybe also by a progressive reduction of SARS-CoV-2 lethality, in line with the classic evolutionary behaviour of these pathogens, as proposed by Hanjun Zhao [20].

An interesting finding of our study, as mentioned, is the observation of a direct, positive correlation of the duration of the hospital admission with the chance of survival during the first wave; while an inverse correlation was observed in the second/third wave.

This result could be explained by the fact that during the first wave patients, who did not survive, died on average at the 6th day of hospital stay (an “early discharge” in comparison with the survivors), while in the second and third wave patients died on mean at the 18th day of hospitalisation (a “longer discharge” in comparison with the survivors). Moreover, the duration of hospitalisation was significantly shorter during the first wave in comparison with second/third wave (10 vs. 15 days, Table 1). Therefore, it could be assumed that during the first wave people died due to COVID-19 respiratory failure, 12 ± 2 days from symptoms onset, in accordance with the most recent literature [1], while in the second and third wave, deaths have been more delayed during the hospital stay, possibly thanks to an earlier hospital admission and to a more prompt and proper treatment; these death are probably to be ascribed mainly to hospital complications rather than a rapid progression of SARS-CoV-2-related lung involvement.

However, although the different timing of deaths distribution between the 1st and 2nd /3rd, no significant differences in hospital-stay complications were detected in our data (Table 3). Therefore, it can be assumed that the deaths ascribable to hospital-stay complications were not significantly different between the 1st and 2nd /3rd wave. Therefore, to explain such a significant decrease in the death toll between the 1st and 2nd /3rd wave, more factors are involved, in particular among them, according to the authors, possibly a reduction of the SARS-CoV-2 virulence.

In agreement with the studies by Luo et al. [21] and Giacomelli et al. [14], it was proved that serum CRP level at hospital admission was independently associated with mortality in COVID-19 different waves. Up to date, it has been shown that in pulmonary diseases marked by inflammatory features, a typical raise in serum CRP level takes place in response to inflammatory cytokines such as interleukin (IL)-6, IL-1 or tumoral necrosis factor (TNF)-α [22]. Thus, CRP serum level might effectively represent an outcome predictor in COVID-19, as suggested by Liu et al. [23] and by Rodriguez et al. [13].

This cohort study, therefore, confirms the well-known and demonstrated prognostic role of the CRP serum level, together with the fraction of inspired oxygen (FiO_2_) needed at the hospital admission; both these parameters may be of help in stratifying the risk of death in an unvaccinated population, such as that enrolled in this study.

Previous studies have conformed the role of respiratory parameters, in particular P/F ratio at admission [24] and of comorbidities [10, 24–26] as determinants of outcome during COVID-19. Also, age and gender might be relevant in this regard [25, 27] together with the Brixia radiologic score and the choice of drugs during hospital admission [10]. No significant interference on the outcome was observed by anti-hypertensive drugs, namely ACE inhibitors or angiotensin-receptor blockers on the outcome was observed in our study, as well as in others [25, 26].

### Limitations of the study

This study has some limitations: in a first instance, it is a monocentric survey; then, due to the emergency setting, in the first wave, the number of laboratory examinations performed at admission were relatively limited, and this is also true for respiratory parameters examined and imaging investigations; therefore, the amount of clinical information was limited by the circumstances.

In conclusion, in the first wave patients tended to arrive later at hospital than in the subsequent periods, with a more severe clinical presentation, as suggested by worse respiratory, bio-humoral and imaging data. Therefore, on the basis of our results, it might be hypothesised that a timely hospital admission and an appropriate duration of the hospital stay could have a significant impact on survival. Moreover, the significant decrease in the mortality rates between 1st and 2nd/3rd wave in a non-vaccinated population may suggest a reduction in the SARS-CoV-2 virulence.

Further studies are, however, needed to confirm these preliminary results. Moreover, long-term post-discharge follow-up is mandatory to better assess the morbidity and mortality due to SARS-CoV-2 infection and the long-term sequelae.
